# Effects of thrombospondin-1 (THBS1) and toll-like receptor 4 (TLR-4) levels in the serum and synovial fluid of patients with knee osteoarthritis

**DOI:** 10.5937/jomb0-62485

**Published:** 2026-06-16

**Authors:** Yingjie Xu, Tian Zhen, Liang Mingjin, Xiaoyun Li

**Affiliations:** 1 The First Affiliated Hospital of Soochow University, Department of Orthopaedics, Suzhou City, China; 2 Peking University Shenzhen Hospital, Orthopedic Joint Department, Shenzhen City, China; 3 Nanjing Medical University Fourth Affiliated Hospital, Department of Orthopaedics, Nanjing City, China

**Keywords:** knee osteoarthritis, thrombospondin-1, toll-like receptor 4, diagnostic value, correlation analysis, osteoartritis kolena, trombospondin-1, tolični receptor 4, dijagnostička vrednost, analiza korelacije

## Abstract

**Background:**

To explore the changes in and significance of Thrombospondin-1 (THBS1) and Toll-like receptor 4 (TLR-4) levels in the serum and joint fluid of patients with knee osteoarthritis (KOA).

**Methods:**

184 KOA patients admitted to our hospital between May 2022 and February 2025 were selected as the observation group, and 64 healthy adults who underwent physical examinations at the same hospital during the same period composed the control group. Tumour necrosis factor-<span style="color: rgb(32, 33, 34); font-family: sans-serif; font-size: 16px; background-color: rgb(248, 249, 250);">α</span> (TNF-<span style="color: rgb(32, 33, 34); font-family: sans-serif; font-size: 16px; background-color: rgb(248, 249, 250);">α</span>), interleukin (IL)-1 b, THBS1, and TLR-4 levels in the two groups' serum and joint fluid were compared. The correlations between TNF-<span style="color: rgb(32, 33, 34); font-family: sans-serif; font-size: 16px; background-color: rgb(248, 249, 250);">α</span>, IL-1 b, THBS1, and TLR-4 levels in the serum and synovial fluid of the two groups and the severity of KOA were evaluated, and the clinical value of THBS1 and TLR-4 in the diagnosis of KOA was analysed.

**Results:**

TNF-<span style="color: rgb(32, 33, 34); font-family: sans-serif; font-size: 16px; background-color: rgb(248, 249, 250);">α</span>, IL-1<span style="color: rgb(32, 33, 34); font-family: sans-serif; font-size: 16px; background-color: rgb(248, 249, 250);">β</span>, THBS1, and TLR-4 levels in the observation group's serum and joint fluid were considerably higher than those in the control group (P&lt; 0.05). With increasing Kellgren-Lawrence (K-L) grade, the observation group's serum and joint fluid showed higher levels of TNF-<span style="color: rgb(32, 33, 34); font-family: sans-serif; font-size: 16px; background-color: rgb(248, 249, 250);">α</span>, IL-1<span style="color: rgb(32, 33, 34); font-family: sans-serif; font-size: 16px; background-color: rgb(248, 249, 250);">β</span>, THBS1, and TLR-4. When TNF-<span style="color: rgb(32, 33, 34); font-family: sans-serif; font-size: 16px; background-color: rgb(248, 249, 250);">α</span>, IL-1<span style="color: rgb(32, 33, 34); font-family: sans-serif; font-size: 16px; background-color: rgb(248, 249, 250);">β</span>, THBS1, and TLR-4 levels were compared among K-L grades, statistically significant differences were found (P&lt; 0.05). The K-L grade of the patients was positively correlated with TNF-<span style="color: rgb(32, 33, 34); font-family: sans-serif; font-size: 16px; background-color: rgb(248, 249, 250);">α</span>, IL-1<span style="color: rgb(32, 33, 34); font-family: sans-serif; font-size: 16px; background-color: rgb(248, 249, 250);">β</span>, THBS1, and TLR-4 in the blood and joint fluid (P&lt; 0.05), and the factors of TNF-<span style="color: rgb(32, 33, 34); font-family: sans-serif; font-size: 16px; background-color: rgb(248, 249, 250);">α</span>, IL-1<span style="color: rgb(32, 33, 34); font-family: sans-serif; font-size: 16px; background-color: rgb(248, 249, 250);">β</span>, THBS1, andTLR-4 in the observation group's serum and joint fluidwere positively correlated with each other (P&lt; 0.05). Thesensitivity and specificity of serum THBS1 and TLR-4 forthe diagnosis of KOA were 0.916 and 0.889, and 0.825and 0.865, respectively, and the optimal cutoff values were247.262 and 5.084 ng/mL, respectively. The sensitivityand specificity of THBS1 and TLR-4 in the joint fluid fordiagnosing KOA were 0.945 and 0.932, and 0.985 and0.916, respectively, and the optimal cutoff values were239.745 and 4.620 ng/mL, respectively.

**Conclusions:**

THBS1 and TLR-4 in serum and synovial fluid can serve as potential markers for the clinical diagnosis and assessment of KOA, with high diagnostic efficacy. Moreover, detecting serum THBS1 and TLR-4 is less invasive, less costly, and more acceptable to patients.

## Abstract (sr)

**Uvod:**

Cilj je bio da se ispitaju promene i značaj nivoa trombospondina-1 (THBS1) i toličnog receptora 4 (TLR-4) u serumu i zglobnoj tečnosti kod pacijenata sa osteoartritisom kolena (KOA).

**Metode:**

Odabrano je 184 pacijenta sa KOA hospitalizovanih u našoj bolnici između maja 2022. i februara 2025. godine kao posmatrana grupa, dok su 64 zdrava odrasla lica koja su obavljala sistematske preglede u istoj bolnici u istom periodu činila kontrolnu grupu. Upoređivani su nivoi faktora nekroze tumora-<span style="color: rgb(32, 33, 34); font-family: sans-serif; font-size: 16px; background-color: rgb(248, 249, 250);">α</span> (TNF-<span style="color: rgb(32, 33, 34); font-family: sans-serif; font-size: 16px; background-color: rgb(248, 249, 250);">α</span>), interleukina-1<span style="color: rgb(32, 33, 34); font-family: sans-serif; font-size: 16px; background-color: rgb(248, 249, 250);">β</span> (IL-1<span style="color: rgb(32, 33, 34); font-family: sans-serif; font-size: 16px; background-color: rgb(248, 249, 250);">β</span>), THBS1 i TLR-4 u serumu i zglobnoj tečnosti obe grupe. Procenjene su korelacije između nivoa TNF-<span style="color: rgb(32, 33, 34); font-family: sans-serif; font-size: 16px; background-color: rgb(248, 249, 250);">α</span>, IL-1<span style="color: rgb(32, 33, 34); font-family: sans-serif; font-size: 16px; background-color: rgb(248, 249, 250);">β</span>, THBS1 i TLR-4 u serumu i sinovijalnoj tečnosti i težine KOA, kao i klinička vrednost THBS1 i TLR-4 u dijagnostici KOA.

**Rezultati:**

Nivoi TNF-<span style="color: rgb(32, 33, 34); font-family: sans-serif; font-size: 16px; background-color: rgb(248, 249, 250);">α</span>, IL-1<span style="color: rgb(32, 33, 34); font-family: sans-serif; font-size: 16px; background-color: rgb(248, 249, 250);">β</span>, THBS1 i TLR-4 u serumu i zglobnoj tečnosti posmatrane grupe bili su značajno viši nego u kontrolnoj grupi (P&lt; 0,05). Sa porastom "Kellgren-Lawrence" (K-L) stepena, nivoi TNF-<span style="color: rgb(32, 33, 34); font-family: sans-serif; font-size: 16px; background-color: rgb(248, 249, 250);">α</span>, IL-1<span style="color: rgb(32, 33, 34); font-family: sans-serif; font-size: 16px; background-color: rgb(248, 249, 250);">β</span>, THBS1 i TLR-4 u serumu i zglobnoj tečnosti posmatrane grupe su se povecavali. Pri poređenju nivoa ovih markera među K-L stepenima uočen je statistički značajan trend (P&lt; 0,05). K-L stepen pacijenata bio je pozitivno povezan sa TNF-<span style="color: rgb(32, 33, 34); font-family: sans-serif; font-size: 16px; background-color: rgb(248, 249, 250);">α</span>, IL-1<span style="color: rgb(32, 33, 34); font-family: sans-serif; font-size: 16px; background-color: rgb(248, 249, 250);">β</span>, THBS1 i TLR-4 u krvi i zglobnoj tečnosti (P&lt; 0,05), a ovi faktori u serumu i zglobnoj tečnosti posmatrane grupe bili su međusobno pozitivno povezani (P&lt; 0,05). Senzitivnost i specifičnost seruma THBS1 i TLR-4 za dijagnozuKOA iznosila je 0,916 i 0,889, odnosno 0,825 i 0,865, aoptimalne granične vrednosti bile su 247,262 i 5,084ng/mL. Senzitivnost i specifičnost THBS1 i TLR-4 u zglobnoj tečnosti za dijagnozu KOA iznosila je 0,945 i 0,932,odnosno 0,985 i 0,916, a optimalne granične vrednostibile su 239,745 i 4,620 ng/mL.

**Zaključak:**

THBS1 i TLR-4 u serumu i sinovijalnoj tečnosti mogu služiti kao potencijalni markeri za kliničku dijagnostiku i procenu KOA, sa visokom dijagnostičkom efikasnošću. Detekcija serumskih markera je manje invazivna, jeftinija i prihvatljivija za pacijente.

## Introduction

Knee osteoarthritis (KOA) is an essential type of osteoarthritis that accounts for approximately 80% of all osteoarthritis cases, and middle-aged and older adults are more likely to have it [1][2][3]. Patients with KOA have degenerative changes in the articular cartilage and significant bone hyperplasia, which are accompanied by synovitis of the bone. This disease is a common chronic inflammatory disease in clinical orthopaedics [4]. In recent years, with the intensification of population ageing, KOA has become the primary orthopaedic disease causing disability among middle-aged and elderly individuals in China. At present, the pathogenesis of KOA is not fully understood [5][6][7]. However, multiple studies have shown that various cytokines, including interleukins (ILs), are involved in the onset and progression of KOA. The Toll-like receptor family can effectively regulate non-specific immunity [8][9][10]. Toll-like receptor 4 (TLR-4) is an important member of the Toll family. TLR-4 is a type I transmembrane protein receptor and is involved in the occurrence of various inflammatory diseases [11]. Recent studies [12][13][14] have shown that TLR-4 is involved in the development and progression of KOA through multiple pathways, including the stimulation of inflammatory mediator secretion and the induction of autophagy [15]. Thrombospondin-1 (THBS1) is an extracellular matrix glycoprotein with multiple biological functions, including regulating platelet aggregation, promoting thrombosis and angiogenesis, and participating in inflammatory responses to various tissue injuries and lesions. Abnormal levels of THBS1 can be detected in the serum of patients with KOA [16].

Nevertheless, as the correlations between KOA patients' blood and synovial fluid levels of TLR-4 and THBS1, and those with other disorders, remain unclear, this study explored the relationships between THBS1 and TLR-4 levels and KOA, aiming to provide a basis for early identification and to guide clinical diagnosis and treatment.

## Materials and methods

### General information

A review of the clinical information of 184 KOA patients who were admitted to our hospital (hereinafter referred to as »our hospital«) from May 2022 to February 2025 was conducted. These patients composed the observation group, and another 80 healthy adults who underwent physical examinations at our hospital during the same period composed the control group. There were 90 females and 94 males in the observation group. The average age was 45.59±6.46 years, with a range of 39 to 62 years. The body mass index (BMI) ranged from 21.13 to 26.45 kg/m^2^, with an average of 23.25±1.56 kg/m^2^. The average duration of the sickness was 4.95±1.69 years, with a range of 1 to 8 years.

In the control group, 80 males and 80 females were included. The age ranged from 35 to 65 years, with an average of 46.13±6.87 years. The BMI ranged from 21.45 to 26.15 kg/m^2^, with an average of 23.13±1.46 kg/m^2^. There were no statistically significant differences in sex, age, or BMI between the two research groups (P>0.05), and the groups were comparable.

### Inclusion and exclusion criteria

Inclusion criteria: (1) According to the »Guidelines for the Diagnosis and Treatment of Osteoarthritis (2022 Edition)« developed by the International Medical Association's Orthopaedics Branch, meet the diagnostic requirements for KOA; (2) Complete clinical medical record materials; (3) The relevant laboratory examination materials are complete.

Exclusion criteria: (1) Those with concurrent knee joint tumors, rheumatoid arthritis, ankylosing spondylitis, etc. (2) Those with a history of knee joint surgery; (3) Those with severe osteoporosis; (4) Those accompanied by local or systemic inflammation, infection and other autoimmune diseases; (5) Those who have a history of using glucocorticoids within one month before the visit.

### Detection methods

Five millilitres of elbow venous blood were drawn from every participant. Following its spontaneous coagulation, the supernatant was collected for subsequent use and centrifuged for 20 minutes at 3,000 r/min. Serum tumour necrosis factor-α (TNF-α), IL-1β, THBS1, and TLR-4 levels were measured using an enzyme-linked immunosorbent assay (ELISA) for a double-antibody sandwich. The kit supplier was Shanghai Lianshuo Biotechnology Co., Ltd. After local anaesthesia, approximately 2 mL of synovial fluid was collected from patients in the observation group and placed in an Eppendorf (Ep) tube for testing. The control group recruited volunteers for synovial fluid extraction using a reward-based approach. One to two millilitres of synovial fluid were extracted and placed in EP tubes for testing. The levels of TNF-α, IL-1β, THBS1 and TLR-4 in the joint fluid were detected via ELISA. The kit was purchased from Shanghai Lianshuo Biotechnology Co., Ltd. No relevant tests or results analyses were conducted for those with dry punctures.

### Observation indicators

(1) The serum and synovial fluid levels of TNF-α, IL-1β, THBS1, and TLR-4 in the two subject groups were compared.

(2) Serum and synovial fluid levels of TNF-α, IL-1β, THBS1, and TLR-4 were examined between individuals with various Kellgren-Lawrence (K-L) grades. Level 0: normal; Grade I: suspected narrowing of the joint space, with possible osteophytes; Grade II: suspected narrowing of the joint space with definite osteophytes; Grade III: imaging reveals significant narrowing of the joint space accompanied by moderate osteophytes, partial sclerosis of the subchondral bone, and suspected deformity. Grade IV: Imaging revealed significant narrowing of the joint space accompanied by large osteophytes, severe hardening of the subchondral bone, and a definite deformity.

(3) Analyse the correlations among the factors of TNF-α, IL-1β, THBS1 and TLR-4 in serum and synovial fluid, as well as between each factor and the severity of KOA. (4) Analyse the clinical value of THBS1 and TLR-4 in the diagnosis of KOA.

### Statistical processing methods

Data analysis was conducted using SPSS 22.0. Measurement data that conform to a normal distribution are expressed as x̄±s. Independent sample t tests were used for comparisons between two groups, one-way analysis of variance was used for comparisons among multiple groups, and pairwise comparisons between groups were conducted using the least significant difference (LSD) test. The χ^2^ test was used to analyse count data, which were expressed as percentages or as the number of cases. Spearman correlation analysis was used to investigate relationships between KOA severity and TNF-α, IL-1β, THBS1, and TLR-4 levels in serum and synovial fluid. To examine relationships among serum and synovial fluid components in KOA patients, Pearson correlation analysis was employed. Using receiver operating characteristic (ROC) curves, the clinical usefulness of THBS1 and TLR-4 in diagnosing KOA was examined. A statistically significant P value was defined as one that was less than 0.05.

## Results

### Comparison of the levels of TNF-α, IL-1β, THBS1 and TLR-4 in the serum and synovial fluid of the two groups of patients

A total of 112 subjects in the control group participated in the determination of joint fluid. Among them, 48 subjects underwent dry puncture or had a small amount of joint fluid sampled and were not included in the analysis. In total, 64 subjects underwent the determination of joint fluid-related factors and analysis of the results. Serum and joint fluid from patients in the observation group had significantly higher levels of TNF-α, IL-1β, THBS1, and TLR-4 than those in the control group (P<0.05) (Table 1).

**Table 1:** Comparison of TNF-α, IL-1β, THBS1, and TLR-4 levels in serum and joint fluid between the two groups of patients (x̄±s).

Group	n	TNF-α (pg/mL)		IL-1β (pg/mL)		THBS1 (ng/mL)		TLR-4 (ng/mL)	
		Serum	Joint fluid	Serum	Joint fluid	Serum	Joint fluid	Serum	Joint fluid
Control <br>group	64	5.52±1.01	7.75±1.69	8.25±1.45	12.18±2.23	213.05±36.68	229.13±41.52	4.26±0.62	3.67±0.76
Observation <br>group	184	51.29±15.65	6155±15.48	58.29±16.47	68.42±16.09	306.94±56.35	351.45±51.63	11.58±3.98	14.55±4.15
t		26.137	19.639	27.143	19.677	12.746	12.095	15.503	14.542
P		<0.001	<0.001	<0.001	<0.001	<0.001	<0.001	<0.001	<0.001

### Comparison of the levels of TNF-α, IL-1β, THBS1 and TLR-4 in the serum and synovial fluid of patients with different K L grades in the observation group

Patients in the observation group had higher serum and joint fluid levels of TNF-α, IL-1β, THBS1, and TLR-4 as their K-L grade rose. TNF-α, IL-1β, THBS1, and TLR-4 levels varied statistically significantly across the various K-L grades (P<0.05).

With the increase in K-L classification, the expression levels of the four factors in serum and synovial fluid increased significantly, and differences among classification groups were statistically significant. Further analysis revealed that the K-L grade of the patients was clearly positively correlated with the increased amplitude of the above factors in serum and synovial fluid. In addition, there is also a significant positive correlation between the levels of TNF-α, IL-1β, THBS1 and TLR-4 in serum and synovial fluid. These results confirm that the expression levels of THBS1 and TLR-4 are closely related to the imaging severity of knee osteoarthritis, and both, together with pro-inflammatory factors, participate in the disease progression (Table 2).

**Table 2:** Comparison of TNF-α, IL-1β, THBS1, and TLR-4 levels in serum and joint fluid of patients with different K-L grades in the observation group (x̄±s).

Project	n	TNF-α (pg/mL)		IL-1β (pg/mL)	
		Serum	Joint fluid	Serum	Joint fluid
Level I	26	26.48±4.27	38.35±3.46	33.25±6.99	44.75±6.18
Level II	42	42.25±6.27	51.32±6.57	45.77±7.02	55.36±7.59
Grade III	64	52.75±6.35	65.82±10.89	63.09±7.74	73.85±7.38
Grade IV	52	69.24±8.58	76.16±8.13	74.99±8.32	84.19±8.88
F		1082.989	390.732	994.655	510.405
P		<0.001	<0.001	<0.001	<0.001
Project	n	THBS1 (ng/mL)		TLR-4 (ng/mL)	
		Serum	Joint fluid	Serum	Joint fluid
Level I	26	249.77±37.75	282.86±22.29	5.15±1.49	6.36±1.09
Level I	42	275.79±53.68	314.52±31.75	11.44±1.93	14.48±2.38
Grade III	64	324.52±26.87	365.18±37.15	12.55±2.66	15.58±2.58
Grade IV	52	339.15±60.66	398.65±28.56	14.26±2.68	17.39±2.14
F		67.407	106.138	192.514	231.119
P		<0.001	<0.001	<0.001	<0.001

### Correlations among various factors in the serum and synovial fluid of the observation group

There was a positive correlation among the factors of TNF-α, IL-1β, THBS1 and TLR-4 in the serum and joint fluid of the observation group (P<0.05).

In patients with knee osteoarthritis, the levels of TNF-α, IL-1β, THBS1 and TLR-4 in serum and synovial fluid show significant synergistic changes. Analysis indicates that there is a clear positive correlation among the four factors from the same body fluid source, indicating that the pro-inflammatory factors and THBS1/TLR-4 form a mutually promoting inflammatory regulatory network in the local microenvironment. Meanwhile, the expression levels of the same factor in serum and synovial fluid are also positively correlated, indicating a pathological link between the systemic circulation and the joint's local microenvironment. These correlations further confirm the mechanism by which THBS1 and TLR-4 collaborate with classical inflammatory factors in the progression of knee osteoarthritis, jointly participating in the disease process and providing a theoretical basis for the joint monitoring of multiple targets (Table 3).

**Table 3:** Correlation analysis between TNF-α, IL-1β, THBS1, and TLR-4 factors in serum and joint fluid.

Source	indicator	IL-1β		THBS1		TLR-4	
		r	P	r	P	r	P
Serum	TNF-α	0.969	<0.001	0.775	<0.001	0.865	<0.001
	IL-1β			0.768	0.001	0.858	<0.001
	THBS1					0.696	<0.001
Joint fluid	TNF-α	0.945	<0.001	0.848	<0.001	0.878	<0.001
	IL-1β			0.859	<0.001	0.875	<0.001
	THBS1					0.799	<0.001

### Correlations between various factors in serum and synovial fluid and the severity of KOA

The levels of TNF-α, IL-1β, THBS1 and TLR-4 in the serum and joint fluid were positively correlated with the K-L grade (P<0.05).

There is a clear positive correlation among the four factors from the same body fluid source, indicating that the pro-inflammatory factors and THBS1/TLR-4 form a mutually promoting inflammatory regulatory network in the local microenvironment. Meanwhile, the expression levels of the same factor in serum and synovial fluid are also positively correlated, indicating a pathological link between the systemic circulation and the joint's local microenvironment. These correlations further confirm the mechanism by which THBS1 and TLR-4 collaborate with classical inflammatory factors in the progression of knee osteoarthritis, jointly participating in the disease process and providing a theoretical basis for the joint monitoring of multiple targets (Table 4).

**Table 4:** Correlation analysis of TNF-α, IL-1β, THBS1, TLR-4 factors in serum and joint fluid with K-L grading.

Source	TNF-α		IL-1β		THBS1		TLR-4	
	r	P	r	P	r	P	r	P
Serum	0.933	<0.001	0.928	<0.001	0.793	<0.001	0.828	<0.001
Joint fluid	0.925	<0.001	0.935	<0.001	0.895	<0.001	0.853	<0.001

### The clinical value of THBS1 and TLR-4 in serum and synovial fluid in the diagnosis of KOA

The sensitivity and specificity of serum THBS1 and TLR-4 for the diagnosis of KOA were 0.913 and 0.887, and 0.826 and 0.863, respectively, and the optimal cutoff values were 247.260 and 5.080 ng/mL, respectively. The sensitivity and specificity of joint fluid THBS1 and TLR-4 for the diagnosis of KOA were 0.948 and 0.937, and 0.989 and 0.906, respectively, and the optimal cutoff values were 239.745 and 4.620 ng/mL, respectively (Table 5).

**Table 5:** Clinical value of THBS1 and TLR-4 in serum and joint fluid for diagnosis of KOA.

Source	Indicator	AUC	Sensitivity	Specificity	Best cutoff value <br>(ng/mL)	95%CI
Serum	THBS1	0.921	0.915	0.889	247.261	0.873~0.967
	TLR-4	0.925	0.827	0.865	5.083	0.872~0.967
Joint fluid	THBS1	0.968	0.949	0.938	239.746	0.941~0.997
	TLR-4	0.989	0.987	0.903	4.622	0.972~1.007

## Discussion

KOA is primarily characterised by degenerative changes in knee cartilage and secondary osteophyte formation [17]. The main clinical signs of this disease are joint deformity, stiffness, and knee pain. It is more common in middle-aged and older adults and is associated with a high disability rate. The occurrence and progression of KOA involve multiple metabolic pathways, including those involved in amino acids, cytokines, and lipids [18]. The markers produced by these pathways are significant for diagnosing, assessing disease, and predicting prognosis in KOA. Inflammatory factors play important roles in KOA progression [19], with changes observed in cytokines, including the interleukin (IL) family and tumour necrosis factor (TNF), during disease development. Among these, IL-1β and TNF-α are considered key contributors to KOA onset, and synovial fluid, synovial membrane, cartilage, and subchondral bone from KOA patients have been found to exhibit notably elevated levels of IL-1β [20][21][22]. In addition, TNF-α can induce the expression of nitric oxide, prostaglandin E2, IL-6 and IL-8 and induce and promote the occurrence and progression of KOA. TNF-α has also been confirmed to be an important biological indicator for the assessment and diagnosis of KOA. Compared with the control group, the observation group's serum and synovial fluid had higher levels of TNF-α and IL-1β, and both were positively associated with the K-L grade, consistent with previous research [23].

THBS1 belongs to the platelet-reactive protein family and has a wide range of physiological functions, including tissue repair, regulation of cell adhesion and migration, promotion of angiogenesis, and promotion of platelet aggregation [24][25][26]. Under normal physiological conditions, THBS1 levels are relatively low. THBS1 levels in the body's tissues and blood, however, can rise significantly if there is a lesion or injury that triggers an inflammatory response. Findings from this investigation showed that the observation group's serum and synovial fluid had higher THBS1 levels than those of the control group [27]. Moreover, with increasing K-L grade, THBS1 levels increased significantly, suggesting that THBS1 levels in serum and synovial fluid play a role in the diagnosis of KOA patients. Additionally, this investigation showed that the K-L grade and the levels of THBS1 in patients' serum and synovial fluid were positively correlated, as were those of TNF-α and IL-1β, suggesting that, with the progression of KOA, THBS1 levels in serum and synovial fluid gradually increased. The cause is that the THBS1/TGF-β1 signalling pathway stimulates an inflammatory response by activating TGF-β1 [28].

Toll-like receptors are transmembrane protein receptors that can specifically initiate autoimmune responses and thereby participate in the occurrence of various diseases. The study's findings showed that the observation group's serum and synovial fluid had higher TLR-4 levels than those of the control group, indicating that TLR-4 levels in serum and synovial fluid have potential value as clinical diagnostic markers of KOA [29]. Similarly, TLR-4 levels increase with higher K-L grade and are significantly positively correlated with K-L grade. It is also positively correlated with TNF-α and IL-1β levels. This may be because the signal transduction pathway of TLR-4 activates the release of cartilage decomposers and various inflammatory factors, such as IL-1β and matrix metalloproteinases (MMPs) [30]. This further leads to or aggravates abnormal cartilage metabolism in patients, inducing or promoting KOA.

In this study, the diagnostic efficacy of THBS1 and TLR-4 levels in serum and synovial fluid for KOA was evaluated using receiver operating characteristic (ROC) curves. The efficacy of THBS1 and TLR-4 in the synovial fluid for the diagnosis of KOA is significantly greater than that in the serum, which is considered closely related to the direct inflammatory response and exudation in the joint space [31][32][33]. However, as an invasive method, joint puncture not only increases local pain and other discomfort when the patient's joint effusion is not very obvious but also poses a risk of damaging the internal tissues of the joint. Owing to patient fear, the method of joint fluid extraction via joint puncture for early KOA diagnosis is likely to be challenging to popularise. Current research [34] has confirmed that various cytokines and markers, including type II collagen carboxyl-terminal peptides and MMPs, can enter the peripheral blood circulation from bones and joints. Therefore, serum markers have obvious advantages in the early diagnosis and assessment of the severity of KOA. Moreover, the sensitivity and specificity of patient serum THBS1 for KOA diagnosis were 0.913 and 0.887, respectively, with an optimal cutoff of 247.26 ng/mL. Furthermore, the results of the ROC curve analysis in this study suggest that serum levels of THBS1 and TLR-4 also have high diagnostic efficacy for diagnosing KOA and assessing disease severity [35]. Moreover, these methods are less invasive, less costly, and more acceptable to patients.

## Conclusion

The levels of THBS1 and TLR-4 in serum and synovial fluid can be used as potential markers for the clinical diagnosis and assessment of KOA conditions, both of which have high diagnostic efficacy. Moreover, detecting serum THBS1 and TLR-4 is less invasive, less costly, and more acceptable to patients.

## Dodatak

### Conflict of interest statement

All the authors declare that they have no conflict of interest in this work.
